# Subclinical diastolic dysfunction and its association with disease activity in patients with rheumatoid arthritis

**DOI:** 10.1007/s11739-026-04413-y

**Published:** 2026-06-23

**Authors:** Christiane Aguiar Nobre, Carlos Ewerton Maia Rodrigues, Luzia Keyne Sousa Carneiro Frota, Natacha Xavier Cavalcante, Thácilla Siqueira Eugênio Nascimento, João Gabriel Marques Brayner, Giovanna Azevedo Sousa, Paula Goes, Vicente de Paulo Teixeira Pinto, Hellíada Vasconcelos Chaves, Mirna Marques Bezerra

**Affiliations:** 1 Postgraduate Program in Translational Medicine, Federal University of Ceará, Fortaleza, Ceará Brazil; 2 School of Medicine, Federal University of Ceará, Sobral, Ceará Brazil; 3 School of Medicine, University Center INTA - UNINTA, Sobral, Ceará Brazil; 4 Graduate Program in Medical Sciences, Medical School, University of Fortaleza (Unifor), Fortaleza, Brazil; 5 Department of Internal Medicine, Federal University of Ceará, Fortaleza, Brazil; 6 Rheumatology Division, Hospital Geral de Fortaleza, Fortaleza, Brazil; 7 Hospital Do Coração, Sobral, Ceará Brazil; 8 Department of Morphology, School of Medicine, Postgraduate Program in Morphological Science, Federal University of Ceará, Fortaleza, Ceará Brazil; 9 Department of Pathology and Legal Medicine, Medical School, Federal University of Ceará, Fortaleza, Ceará Brazil; 10 School of Dentistry, Federal University of Ceará, Sobral, Ceará Brazil

**Keywords:** Rheumatoid arthritis, Disease activity, Cardiovascular risk, Ultrasonography, Echocardiogram, Subclinical diastolic dysfunction

## Abstract

Rheumatoid arthritis (RA) is a chronic inflammatory disorder associated with cardiovascular risk (CVR), regardless of traditional risk factors. The objective of this study was to assess subclinical cardiovascular changes in RA, including cardiac and carotid parameters, and to examine their correlation with disease activity in 60 RA patients and 60 age and sex-matched healthy controls. The clinical variables included epidemiological and laboratory data and disease activity scores (DAS28-PCR). CVR was stratified using SCORE/mSCORE and Framingham scores. The ultrasound (US) methods employed (transthoracic echocardiography and Doppler carotid ultrasonography) allowed to evaluate carotid intima–media thickness (CIMT), atherosclerosis plaque, and cardiac function. The female sex was predominant (RA = 91.7%; controls = 90%) and the mean age was 52 ± 12 years and 52 ± 13 years, respectively. The mean disease duration was 10.43 ± 7.55 years. Serum testing for rheumatoid factor and anti-CCP was double-negative in 36.7%. The DAS28-PCR scores identified 61.7% as ‘remission/low activity’ and 31.3% as ‘moderate/high activity’. CVR was similar in the two groups (*p* = 0.261). When the echocardiographic data was stratified, diastolic dysfunction was significantly more prevalent in subjects with higher disease activity (*p* = 0.04). In addition, high clinical CVR scores were associated with greater CIMT and carotid plaque (*p* < 0.05). Our results suggest that inflammatory activity may play a major role in subclinical cardiovascular dysfunction in RA.

## Introduction

A chronic inflammatory condition affecting the joints, rheumatoid arthritis (RA) is considered an independent cardiovascular risk factor (CVRF) [1, 2]. As shown in the literature, RA patients are at a significantly higher risk of cardiovascular events and mortality than the general population [3, 4], mostly due to persistent systemic inflammation.

Disease activity in patients with RA is directly related to cardiovascular risk, and this risk is proportional to the intensity and persistence of systemic inflammation, which contributes to endothelial dysfunction, lipid alterations, and a higher prevalence of subclinical atherosclerotic plaques [5–7].

The repercussion of RA on the cardiovascular system are multifaceted and generally involve structural and functional changes to the heart [8–10]. Functional changes often include diastolic dysfunction and reduced ejection fraction [11, 12].

An understanding of the negative impact of cardiovascular disease (CVD) on overall health, quality of life and life expectancy, as well as of the early detection of subclinical signs, is essential for risk stratification and management of RA patients. Ultrasonography (US) is usually employed to this end, but the ideal method has not yet been established [13]. US modalities like transthoracic echocardiography and Doppler carotid ultrasonography are low-cost, non-invasive, and sensitive tools in cardiovascular screening [5, 9]. In fact, a study on 104 RA patients found carotid US to be more sensitive than coronary artery calcium scores in the detection of subclinical atherosclerosis in RA [14].

The combination of traditional clinical scores with cardiac and carotid imaging methods is recommended to enhance cardiovascular risk assessment. The populations that benefit most from integrating traditional clinical scores with cardiac and carotid imaging methods for cardiovascular risk assessment are asymptomatic adults classified as low to intermediate risk by traditional scores (such as Framingham, SCORE), particularly those aged 40–65 years, without known cardiovascular disease [15–17].

In view of the known association between disease activity and increased CVRF in patients with RA, the objective of this study was to assess subclinical cardiovascular changes in RA, including cardiac and carotid parameters, and to examine their correlation with disease activity in patients from Northeastern Brazil.

## Methods

### Patients

This cross-sectional study was conducted at a secondary-level healthcare facility in Northeastern Brazil between October 2021 and October 2022. The study protocol complied with the principles of the Declaration of Helsinki [18] and was approved under #10,729/2020 by the research ethics committee of the Federal University of Ceará (CEP/UFC/PROPESQ). All procedures followed well-established research standards and guidelines, and all participants gave their informed written consent prior to entering the study.

The sample consisted of 120 participants, of whom half were RA patients and half were sex and age-matched healthy controls recruited from the local community. All RA patients met the 2010 diagnostic criteria of the American College of Rheumatology [19]. The exclusion criteria were age under 18 years, pregnancy, previously diagnosed CVD (including atrial fibrillation), chronic kidney failure, and association with collagen disorders such as systemic lupus erythematosus, Sjögren syndrome, inflammatory myopathy, and sclerodermia.

### Sample size calculation

The sample size was calculated considering carotid intima–media thickness (CIMT) as primary variable outcome. In an earlier study [20], patients with recent-onset RA had an average CIMT of 0.64 ± 0.13 mm, compared to 0.58 ± 0.09 mm in healthy controls. Based on this expected difference, we estimated that a statistical power of 80% at the 5% level of significance (α = 0.05) would require at least 55 participants per group. In this study, each group included 60 subjects, matching the design adopted in another cross-sectional study [21].

### Clinical and anthropometric evaluation

The collected information included epidemiological, clinical, and laboratory data, such as blood count, C-reactive protein, rheumatoid factor (RF), anti-cyclic citrullinated peptide antibody (anti-CCP), glycemia, total cholesterol, high-density lipoprotein (HDL-c), low-density lipoprotein (LDL-c), and triglycerides.

The 10-year cardiovascular risk was assessed using the systematic coronary risk evaluation (SCORE), the modified SCORE (mSCORE), and the Framingham score [22, 23]. To calculate mSCORE, we multiplied the SCORE index by 1.5 in RA patients meeting at least 2 of the following 3 criteria: disease duration > 10 years, positivity for FR and/or anti-CCP, and extra-articular manifestations [24].

Upon physical examination, information was collected on the number of tender and swollen joints, arterial blood pressure, waist circumference (WC), and body mass index (BMI). WC was measured with a tape positioned horizontally between the iliac crest and the lowest rib. BMI was calculated by dividing the weight by the height squared (kg/m^2^). Overweight was defined as BMI 25.0–29.9 kg/m^2^ and obesity was defined as ≥ 30.0 kg/m^2^.

Disease activity was scored with the composite index DAS28-CRP, using the following interpretation: < 2.6 = clinical remission; 2.6–3.1 = low activity; 3.2–5.0 = moderate activity; > 5.1 = high activity [25].

### Ultrasound evaluation

Both groups of participants were submitted to cardiac and carotid US by a cardiologist (LKSCF) trained and experienced in the respective US techniques. Carotid US in B-mode with spectral Doppler allowed to conduct a morphological and hemodynamic analysis, with emphasis on CIMT measurement and the detection of atherosclerosis plaque―a well-established marker of subclinical atherosclerosis [26, 27]. Cardiac function was evaluated by 2-dimensional transthoracic echocardiography while the left ventricle ejection fraction (LVEF) was determined with the Teicholz method, following the guidelines of the American Society of Echocardiography [28].

### Statistical analysis

Clinical and demographic variables, when quantitative and continuous, were expressed as means ± standard deviation (SD) and medians + interquartile range, or, when categorical, as absolute numbers (n) and percentages (%). The main groups (RA vs. controls) and the CVRF subgroups were compared with the chi-squared test (categorical variables) or the Mann–Whitney and Kruskal–Wallis test (non-parametric and continuous variables). All statistical analyses were performed with the software SPSS v. 26.0 (IBM Corp., Armonk, NY, USA) at the 5% level of significance (*p* < 0.05).

## Results

The female sex was predominant in both the RA group (91.7%) and the control group (90%). The mean age was 52 ± 12 years and 52 ± 13 years, respectively.

In the case–control analysis, when comparing the groups with regard to traditional CVRFs (family history of CVD, current or previous smoking, arterial hypertension, diabetes mellitus, sedentary lifestyle, systolic and diastolic blood pressure, total cholesterol, HDL and LDL), no significant difference was observed (*p* > 0.05). Likewise, the two groups were statistically similar with regard to BMI and the distribution of low weight, overweight and obesity, but the prevalence of dyslipidemia was significantly higher in the control group (55.0%) than in the RA group (33.3%) (*p* = 0.027). Moreover, the controls displayed higher WC (91.67 cm vs. 87.65 cm; *p* = 0.017) and BMI values (28.58 vs. 26.83; *p* = 0.010). As for laboratory parameters, the controls had significantly higher levels of glycemia (109.95 mg/dL vs. 93.92 mg/dL; *p* < 0.001) and triglycerides (154.27 mg/dL vs. 114.37 mg/dL; *p* = 0.004).

The mean disease duration was 10.43 ± 7.55 years, distributed as follows: 1–5 years = 30%, 5–10 years = 18.3%, and > 10 years = 51.7%. The mean time since diagnosis was 8.57 ± 6.84 years. Positivity for either RF or anti-CCP was 20%, for both 43.3%, and for neither 36.7%. According to the DAS-28 PCR scoring system, 61.7% were classified as remission or low activity. The remainder (31.3%) displayed moderate or high activity.

With regard to therapy, 59 of the 60 patients (98.3%) were treated with disease-modifying antirheumatic drugs (DMARDs), csDMARDs (conventional synthetic DMARDs) (n = 55; 91.7%) used alone (n = 39; 66.1%) or in combination with biological DMARDs (n = 13; 22.0%) or targeted synthetic DMARDs (n = 7; 11.9%). Current use of corticosteroids was reported by 26.7%, while methotrexate was used by 72.9%.

Table 1 shows the CVRFs of participants with and without RA expressed in SCORE/mSCORE and Framingham scores. According to the SCORE/mSCORE index, low risk was assigned to 45% of the RA patients and to 55.4% of the controls. Moderate risk was higher among RA patients (55% vs. 44.6%), but the difference was not significant (*p* = 0.261). The mean Framingham score was slightly higher in the control group (8.34 ± 8.95%) than in the RA group (6.33 ± 6.78%), though not significantly (*p* = 0.208). When arranged by category, low CVR (≤ 10%) was assigned to 68.3% of the controls and to 85% of the RA patients. Moderate risk (10–20%) and high risk (≥ 20%) were less frequent in the RA group, though not significantly. No cases of atrial fibrillation were identified during clinical evaluation or echocardiographic assessment upon inclusion in the study.

**Table 1:** Cardiovascular risk factors (CVRFs) determined by ultrasound scanning of subjects without (controls) and with rheumatoid arthritis (RA)

Variables	Controls (n = 60)	RA (n = 60)	*p*-value
Ultrasound findings			
Intima–media thickness R (mm)	0.65 ± 0.15	0.66 ± 0.14	0.346^b^
Intima–media thickness L (mm)	0.65 ± 0.15	0.68 ± 0.19	0.474^b^
Plaque D			
Yes	8 (13.3%)	6 (10%)	0.777^a^
No	52 (86.7%)	54 (90%)	
Plaque L			
Yes	4 (6.7%)	8 (13.3%)	0.362^a^
No	56 (93.3%)	52 (86.7%)	
Teicholz ejection fraction	65.92 ± 5.55	64.34 ± 5.35	0.097^b^
Diastolic dysfunction			
Yes	14 (23.3%)	8 (13.3%)	0.239^a^
No	46 (76.7%)	52 (86.7%)	

Data expressed as means ± standard deviation of the mean, or absolute numbers (n) and percentages (%). mm = millímeters. ^a^ Pearson’s Chi-squared test. ^b^ Mann–Whitney test.

Cardiac and carotid US findings were statistically similar in the two groups with regard to CVR parameters (Table 1). However, when the RA group was further stratified according to pharmacological therapy, distinct differences in cardiac function emerged. Specifically, patients receiving bDMARDs had significantly lower left ventricular ejection fractions, while those treated with csDMARDs displayed higher values. No significant differences were observed for corticosteroid or tsDMARD use (Table 2).

**Table 2:** Association between pharmacological therapy and cardiac and carotid ultrasound parameters in patients with RA

Therapy	Yes (mean ± SD)	No (mean ± SD)	*p*-value
Teicholz ejection fraction (%)			
Corticosteroids	64.56 ± 4.00	64.26 ± 5.80	0.446
Only csDMARDs	65.35 ± 5.34	62.71 ± 5.06	0.040a
bDMARDs	61.88 ± 4.64	65.02 ± 5.38	0.042a
tsDMARDs	0.72 ± 0.31	0.67 ± 0.17	0.822
Intima–media thickness R (mm)			
Corticosteroids	0.69 ± 0.13	0.65 ± 0.15	0.366
Only csDMARDs	0.64 ± 0.12	0.70 ± 0.17	0.330
bDMARDs	0.69 ± 0.11	0.65 ± 0.15	0.167
tsDMARDs	0. 75 ± 0.26	0.65 ± 0.12	0.527
Intima–media thickness L (mm)			
Corticosteroids	0.65 ± 0.11	0.69 ± 0.21	0.751
Only csDMARDs	0.64 ± 0.12	0.74 ± 0.25	0.188
bDMARDs	0.74 ± 0.22	0.66 ± 0.18	0.110
tsDMARDs	0.72 ± 0.31	0.67 ± 0.17	0.822

Data expressed as means ± standard deviation of the mean. mm = millimeters; DMARDs = disease-modifying antirheumatic drugs; csDMARDs = conventional synthetic DMARDs; bDMARDs = biological DMARDs; tsDMARDs = targeted synthetic DMARDs; R = right; L = left. ^a^ = Mann–Whitney test. Bold type = significant at 5% (*p* < 0.05).

Within the RA group, the DAS28-PCR scores were statistically similar for remission/low activity and moderate/high activity, left and right side CIMT, and atherosclerosis plaque (Table 3), but diastolic dysfunction was more prevalent in patients with moderate/high activity (33.3%) than in patients with remission/low activity (5.7%) (*p* = 0.044).

**Table 3:** DAS28-PCR scores versus US findings in subjects with rheumatoid arthritis (RA) (n = 60)

Variables	DAS28-PCR		*p*-value
	Remission/Low	Moderate/High	
Echocardiographic findings			
Intima–media thickness R (mm)	0.66 ± 0.14	0.67 ± 0.15	0.837^b^
Intima–media thickness L (mm)	0.63 ± 0.11	0.75 ± 0.25	0.058^b^
Plaque R (n/%)			
Yes	4 (10.8)	2 (8.7)	1.000^a^
No	33 (89.2)	21 (91.3)	
Plaque L (n/%)			
Yes	5 (13.5)	3 (13.0)	1.000^a^
No	32 (86.5)	20 (87.0)	
Teicholz ejection fraction	63.33 ± 4.56	65.96 ± 6.18	
Diastolic dysfunction (n/%)			
Yes	2 (5.7)	8 (33.3)	**0.044**^**a**^
No	35 (94.3)	16 (66.7)	

Data expressed as means ± standard deviation of the mean, or absolute numbers (n) and percentages (%). mm = milliímeters. ^a^ = Pearson’s chi-squared test. ^b^ = Mann–Whitney test. Bold type = significant at 5% (*p* < 0.05).

When comparing low and moderate CVR in RA patients expressed in SCORE/mSCORE, some echocardiographic parameters differed significantly (Table 4). Thus, left and right-side CIMT values were significantly higher for moderate risk (0.69 mm and 0.71 mm; *p* = 0.048) than for low risk (0.62 mm and 0.60 mm; *p* < 0.001). Atherosclerosis plaque in the right carotid artery was also more common for moderate risk (14.5%) than for low risk (3.4%) (*p* = 0.049). Finally, diastolic dysfunction was significantly more frequent among participants with moderate risk (32.1% vs. 5.2%; *p* < 0.001).

**Table 4:** Association between echocardiographic findings and cardiovascular risk expressed in SCORE/mSCORE in subjects with rheumatoid arthritis (RA) (n = 60)

Variables	SCORE/mSCORE scores		*p*-value
	Low	Moderate	
Echocardiographic findings			
Intima–media thickness R (mm)	0.62 ± 0.12	0.69 ± 0.16	**0.048**^**b**^
Intima–media thickness L (mm)	0.60 ± 0.11	0.71 ± 0.18	** < 0.001**^**b**^
Plaque R (n/%)			
Yes	2 (3.4)	8 (14.5)	**0.049**^**b**^
No	56 (96.6)	47 (85.5)	
Plaque L (n/%)			
Yes	2 (3.4)	7 (12.7)	0.089^a^
No	56 (96.6)	48 (87.3)	
Teicholz ejection fraction Diastolic dysfunction (n/%)	64.92 ± 5.77	64.92 ± 5.77	0.538^b^
Yes	3 (5.2)	17 (32.1)	** < 0.001**^**b**^
No	55 (94.8)	36 (67.9)	

Data expressed as mean ± standard deviation of the mean, or absolute numbers (n) and percentages (%). mm=millimeters ^a^=Pearson’s Chi-squared test. ^b^=Mann–Whitney test. Bold type=significant at 5% (*p*<0.05)

The Framingham scores assigned to the echocardiographic findings of the RA patients (Table 5) reflected major differences in mean CIMT, which was significantly greater for moderate/high risk on the right side (0.70 mm) and the left side (moderate 0.91 mm; high 0.74 mm) than for low risk (both 0.65 mm) (*p* = 0.042 and *p* < 0.011, respectively). Atherosclerosis plaque in the carotids was also significantly more frequent in high-risk patients. Despite these structural changes, the ejection fraction and the prevalence of diastolic dysfunction were similar in the two groups (*p* > 0.5).

**Table 5:** Association between echocardiographic findings and cardiovascular risk expressed in Framingham scores in subjects with rheumatoid arthritis (RA) (n = 60)

Variables	Framingham scores			*p*-value
	Low	Moderate	High	
Echocardiographic findings				
Intima–media thickness R (mm)	0.65 ± 0.15	0.7 ± 0.1	0.7 ± 0.03	**0.042**^**b**^
Intima–media thickness L (mm)	0.65 ± 0.17	0.91 ± 0.26	0.74 ± 0.19	** < 0.011**^**b**^
Plaque L (n/%)				
Yes	2 (3.9)	2 (40.0)	2 (50.0)	**0.009**^**b**^
No	49 (96.1)	3 (60.0)	2 (50.0)	
Plaque L (n/%)				
Yes	5 (9.8)	3 (60.0)	0 (0.0)	**0.020**^**a**^
No	46 (90.2)	2 (40.0)	4 (100.0)	
Teicholz ejection fraction Diastolic dysfunction (n/%)	64.22 ± 5.57	65.8 ± 2.28	64 ± 5.89	0.505^b^
Yes	7 (14.0)	0 (0.0)	1 (25.0)	< 0.867^b^
No	43 (86.0)	4 (100.0)	3 (75.0)	

Data expressed as means ± standard deviation of the mean, or absolute numbers (n) and percentages (%). mm=millímeters. ^a^=Pearson’s Chi-squared test. ^b^=Mann–Whitney test. Bold type=significant at 5% (*p*<0.05)

No significant association was observed between the echocardiograhic parameters and disease duration (Table 6), or between US changes and positivity for RF and/or anti-CCP (Table 7).

**Table 6:** Association between echocardiographic findings and disease duration in subjects with rheumatoid arthritis (RA) (n = 60)

Variables	Disease duration			*p*-value
	1–5 years	5–10 years	> 10 years	
Echocardiographic findings				
Intima–media thickness R (mm)	0.63 ± 0.12	0.70 ± 0.11	0.67 ± 0.16	0.263^b^
Intima–media thickness L (mm)	0.64 ± 0.11	0.67 ± 0.11	0.70 ± 0.24	0.569^b^
Plaque R (n/%)				
Yes	1 (5.6)	1 (9.1)	4 (12.9)	0.849^b^
No	17 (94.4)	10 (90.9)	27 (87.1)	
Plaque L (n/%)				
Yes	1 (5.6)	2 (18.2)	5 (16.1)	0.596^a^
No	17 (94.4)	9 (81.8)	26 (83.9)	
Teicholz ejection fraction Diastolic dysfunction (n/%)	64.18 ± 4.98	63.64 ± 6.67	64.68 ± 5.21	0.733^b^
Yes	2 (11.1)	4 (36.4)	4 (12.9)	0.352^a^
No	16 (88.9)	7 (63.6)	27 (87.1)	

Data expressed as means ± standard deviation of the mean, or absolute numbers (n) and percentages (%). mm=millimeters ^a^=Pearson’s Chi-squared test. ^b^=Kruskal–Wallis test followed by Dunn’s post hoc test. Bold type=significant at 5% (*p*<0.05)

**Table 7:** Association between serum profile (rheumatoid factor and anti-CCP) and echocardiographic findings in subjects with rheumatoid arthritis (RA) (n = 60)

Variables	RF + and anti-CCP +	RF + or anti-CCP +	RF − and anti-CCP −	*p*-value
Echocardiographic findings				
Intima–media thickness R (mm)	0.70 ± 0.16	0.63 ± 0.11	0.64 ± 0.14	0.307^b^
Intima–media thickness L (mm)	0.68 ± 0.18	0.62 ± 0.11	0.71 ± 0.22	0.537^b^
Plaque R (n/%)				
Yes	2 (7.7)	1 (8.3)	3 (13.6)	0.856^b^
No	24 (92.3)	11 (91.7)	19 (86.4)	
Plaque L (n/%)				
Yes	3 (11.5)	2 (16.7)	3 (13.6)	1.000^b^
No	23 (88.5)	10 (83.3)	19 (86.4)	
Teicholz ejection fraction	65.05 ± 5.38	63.58 ± 6.01	63.91 ± 5.09	0.660^b^
Diastolic dysfunction (n/%)				
Yes	5 (19.2)	1 (8.3)	3 (14.3)	0.895^a^
No	21( 80.3)	11 (91.7)	18 (85.7)	

Data expressed as means ± standard deviation of the mean, or absolute numbers (n) and percentages (%). mm = millimeters ^a^ = Pearson’s Chi-squared test. ^b^ = Kruskal–Wallis test followed by Dunn’s post hoc test. Bold type = significant at 5% (*p* < 0.05).

## Discussion

In this study, we investigated cardiac and carotid functional and structural changes in RA patients and their association with disease activity, clinical findings, and cardiovascular risk. The clinical SCORE/mSCORE and Framingham scores were statistically similar for RA patients and controls, but RA patients with high scores displayed changes on US, such as increased CIMT and prevalence of carotid atherosclerosis, suggesting subclinical vascular impairment.

Despite the absence of statistical significance between RA patients and controls with regard to overall cardiac and carotid US parameters, when we stratified the data the results proved to be relevant. Thus, the fact that diastolic dysfunction was significantly more frequent in RA patients with moderate/high disease activity suggests an association between systemic inflammation and functional cardiac impairment. Moreover, subjects with moderate CVR also tended to have more severe arterial thickening, carotid plaque and diastolic dysfunction. These findings are consistent with published evidence that 30% of cardiovascular risk in RA patients is due to the disease, while the remaining 70% is explained by traditional CVRFs like hypertension and smoking [29].

In our RA patients, the highest risk scores coincided with US changes suggestive of subclinical atherosclerosis. Clinical CVR scores like Framingham and SCORE are widely used in clinical practice to estimate the 10-year risk of cardiovascular events, but evidence shows that these models are of limited usefulness in patients with autoimmune disease, such as RA, because they do not adequately take into account the role of chronic inflammation in the development of atherosclerosis [30]. Even the correction factor of 1.5 proposed by EULAR seems insufficient to assess the actual risk in this patient population [31]. On the other hand, the evaluation of subclinical atherosclerosis on carotid US―a non-invasive, low-cost, reproducible and sensitive method capable of early detection of structural changes, such as increased CIMT and plaque formation―has been shown to compensate for this shortcoming. In short, combining clinical scores and US findings allows for a more accurate and low-cost risk stratification of RA patients, hence earlier and more targeted interventions [32].

The prevalence of diastolic dysfunction in RA patients is reported to be between 31% and > 50%, depending on population profile and follow-up time. The change is usually detected on Doppler echocardiography, even in patients with preserved ejection fraction and without evident cardiovascular symptoms [33–37]. Disease activity, as expressed by increased DAS28 and CDAI scores and levels of CRP and ESR, is independently correlated with the presence and severity of diastolic dysfunction [33–40]. Patients with active RA are known to be at a significantly higher risk of heart failure, especially diastolic, than patients with RA in remission or low activity [38, 39]. The fact that the prevalence of diastolic dysfunction was similar in healthy controls and in RA patients with low disease activity suggests that careful control of inflammation can mitigate subclinical CVR [40]. The finding in the present study of an association between diastolic dysfunction and high RA activity supports this notion.

Additionally, sex-related biological differences are known to influence cardiovascular structure and function in RA. Although RA predominantly affects women, male patients tend to present a disproportionately higher cardiovascular risk and worse outcomes. In the present study, the small number of male participants precluded meaningful sex-stratified analyses. Moreover, hormonal factors, such as menopausal status, may also impact cardiovascular remodeling and diastolic function. Sex-specific and hormonal influences should therefore be taken into accountwhen interpreting echocardiographic findings in RA populations. Studies with larger and more balanced samples are neededto clarify these associations, as pointed out in therecent literature on sex and gender medicine in cardiovascular disease [41, 42].

The correlation in RA patients between high clinical CVR scores and US findings suggestive of subclinical atherosclerosis has been pointed out elsewhere, but the literature on this specific topic is very limited [43]. Importantly, the association established in our study between cardiovascular changes (diastolic dysfunction, increased CIMT, atherosclerosis plaque) and high clinical CVR scores highlights the need for an integrated approach to CVR in this patient population, combining clinical stratification, US scanning, and monitoring of inflammatory activity [14–16].

No association was found between disease duration and cardiovascular changes. Classically, disease duration has been regarded as an independent predictor of arterial stiffness, since aging of the arteries is more accelerated in RA than in healthy controls [44], and since the cumulative damage caused by RA is greater in patients with ≥ 10 years of disease, even in the absence of traditional CVRFs [45]. Interestingly, neither did we observe any association between positivity for antibodies and changes on US. Cohort studies with long follow-up have shown that both RF and anti-CCP are associated with an increased incidence of cardiovascular events in patients with established RA, including acute coronary syndrome, stroke, and cardiovascular death [46, 47]. The risk is particularly high in subjects with high anti-CCP titers. On the other hand, and supporting our findings, a large cohort study concluded that, if traditional CVRFs and systemic inflammation are adjusted for, positivity for RF or anti-CCP is in itself not a robust, independent predictor of cardiovascular events, with inflammatory activity and disease severity being the main risk determinants [48].

Considering that systemic inflammation is a major factor in the acceleration of atherosclerosis in RA, the absence in our study of a significant association between disease duration, serum positivity, and structural and functional cardiovascular changes may in part be explained by the fact that over 60% of our sample displayed RA in remission/low activity due to efficient clinical control.

Although an association between bDMARD use and lower ejection fraction was observed in our sample, current evidence does not support a direct deleterious effect of biological therapies on cardiac function. On the contrary, several studies suggest potential cardioprotective effects, particularly with TNF inhibitors. Therefore, this finding is more likely explained by confounding by indication, reflecting the greater disease severity and inflammatory burden of patients requiring biological therapy. Furthermore, left ventricular ejection fraction remained within normal ranges across all groups, reinforcing the subclinical nature of the observed differences [49, 50].

The strengths of this study include i) our integrated approach (combining echocardiography and carotid US) to improve the analysis of structural and functional cardiovascular changes and subclinical atherosclerosis, and ii) the combination of US findings with CVR scores to improve the correlation between these parameters and cardiac screening in RA. On the other hand, the study was limited by i) the small sample size, ii) the single-center design, iii) the cross-sectional observational design, iv) the lack of multivariate analysis to identify independent predictors of CVR in RA patients submitted to US, v) the lack of detailed data regarding blood pressure control and antihypertensive treatment, which may have influenced diastolic function in our patients, and vi) the absence of specific structural and functional atrial measurements, precluding a direct assessment of atrial cardiomyopathy. These parameters may provide relevant information, given that systemic inflammation in RA is known to contribute to myocardial and atrial remodeling, potentially preceding clinically overt cardiovascular disease [51]

## Conclusion

In conclusion, our findings showed that inflammatory activity plays a major role in subclinical cardiovascular dysfunction in a sample of RA patients. Hence, US screening of the heart and carotids should be considered for earlier detection and intervention, especially in subjects with active RA or additional CVRFs. Further investigations are needed to explore the methods of early detection of CVRFs in RA.

## Data Availability

The datasets used and/or analysed during the current study are available from the corresponding author on reasonable request.
